# Plant extracts and natural compounds used against UVB-induced photoaging

**DOI:** 10.1007/s10522-017-9715-7

**Published:** 2017-07-12

**Authors:** Maria Cavinato, Birgit Waltenberger, Giorgia Baraldo, Carla V. C. Grade, Hermann Stuppner, Pidder Jansen-Dürr

**Affiliations:** 1 Institute for Biomedical Aging Research, University of Innsbruck, Rennweg 10, 6020 Innsbruck, Austria; 2 Institute of Pharmacy/Pharmacognosy, University of Innsbruck, Innsbruck, Austria; 3 Center for Molecular Biosciences Innsbruck (CMBI), Innsbruck, Austria; 4 Instituto Latino-Americano de Ciências da Vida e da Natureza, Universidade Federal da Integração Latino-Americana, Foz do Iguaçu, Brazil

**Keywords:** Skin aging, UVB, Natural compounds, Plant extracts, Photoaging, Cosmetics

## Abstract

Skin is continuously exposed to a variety of environmental stresses, including ultraviolet (UV) radiation. UVB is an inherent component of sunlight that crosses the epidermis and reaches the upper dermis, leading to increased oxidative stress, activation of inflammatory response and accumulation of DNA damage among other effects. The increase in UVB radiation on earth due to the destruction of stratospheric ozone poses a major environmental threat to the skin, increasing the risk of damage with long-term consequences, such as photoaging and photocarcinogenesis. Extracts from plants and natural compounds have been historically used in traditional medicine in the form of teas and ointments but the effect of most of these compounds has yet to be verified. Regarding the increasing concern of the population with issues related to quality of life and appearance, the cosmetic market for anti-aging and photoprotective products based on natural compounds is continuously growing, and there is increasing requirement of expansion on research in this field. In this review we summarized the most current and relevant information concerning plant extracts and natural compounds that are able to protect or mitigate the deleterious effects caused by photoaging in different experimental models.

## Introduction

**Fig. 1:**
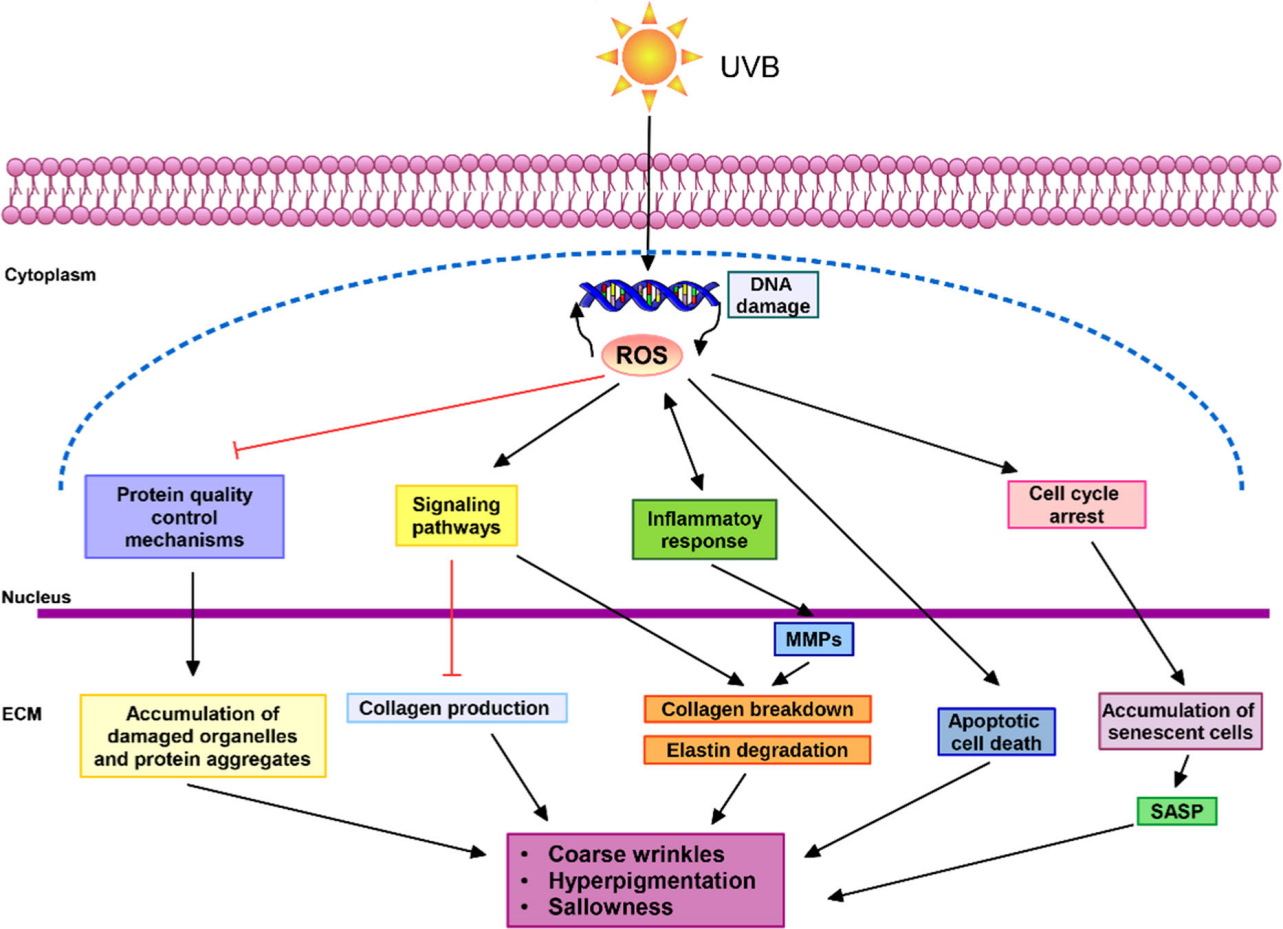
Mechanisms of UVB-induced skin aging. Summary of the main pathways affected by chronical exposure of the skin to UVB and their impact in the appearance of the tissue

Skin is the outermost organ of the body and is subjected to environmental damage such as sunlight and pollution among others. Skin aging is the result of two synergistic mechanisms: intrinsic or chronological aging, a process that occurs not just to the skin but to all tissues and is a result of passage of time; and extrinsic aging, or photoaging, which is caused by repetitive exposure of the skin to damaging agents, especially sunlight (Naylor et al. 2011). UVB is the most dangerous component of sunlight. Due to its high energy, UVB is able to cross the epidermis and reach the upper dermis where is interacts with cellular chromophores, leading to DNA damage and increased oxidative stress (Trautinger 2001; Cavinato and Jansen-Dürr 2017). These events activate innumerous signaling pathways that lead to decreased collagen production, increased synthesis and activity of matrix metalloproteases (MMPs) which are responsible for connective tissue degradation, accumulation of senescent cells, synthesis and accumulation of the senescence-associated secretory phenotype (SASP) components and defective degradation of elastic fibers (Cavinato et al. 2016; Cavinato and Jansen-Dürr 2017) (Fig. 1). Macroscopically, these events result in the appearance of wrinkles, increased epidermal thickness with consequent increased dehydration, hyperpigmentation, sallowness, and loss of skin tone, which are the main characteristics of photoaged skin (Quan et al. 2004). The increment in UVB radiation on earth due to the destruction of the ozone layer, is a major environmental threat to the skin, increasing the risk of damage with long-term consequences, such as photoaging, photoimmunosuppression and photocarcinogenesis (Decean et al. 2016).

In an era that has been witnessing a significant increase in life expectancy and consequent growth of the elderly population, knowledge of physiological changes and diseases most frequently observed in older skin is particularly important (Kim et al. 2013). Moreover, the appearance of elderly skin and the increasing demand for therapeutic interventions to minimize aging manifestations has led to a renewed interest in this matter by researchers, pharmaceutical and cosmetic industries (Draelos 2000). The market for natural cosmetic products is one of the fastest growing in the world and according to a recent survey, the global demand for these personal care products is expected to increase around 9.6% until 2018 (source: Transparent Market Research, http://www.transparencymarketresearch.com/organic-personal-care-products.html). The fields of skincare research and pharmacological characterization of natural compounds are evolving together with the demand of the market (Tundis et al. 2015).

Natural compounds are used for dermatologic purposes both as oral dietary supplements as well as in topical cosmetic formulations (Allemann and Baumann 2009). The vast array of techniques currently available to investigate skin responsivity to multiple stimuli has brought about a new era in cosmetic and dermocosmetic development based on a robust understanding of skin physiology and its varied responses to commonly encountered environmental insults (Dreno et al. 2014). The importance of cosmetic research is not only related to improving the skin overall appearance during aging, but also aims to offer better quality of life acting through prevention and treatment of skin disorders related to the aging process (Kraft and Lynde 2005). Several active ingredients have been identified as regulatory elements of skin homeostasis, with potential cosmetic and/or dermatological applications (Gaspar et al. 2008; Watson et al. 2009; Kanlayavattanakul and Lourith 2010; Anunciato and da Rocha Filho 2012); however, the effectiveness of many formulations has yet to be confirmed. Moreover, considering the diversity of plants and natural compounds available, this field of study has large potential for growth. Given this fact, a discussion of all the currently available literature related to natural compounds and plant extracts used in dermatology would exceed the scope of this manuscript. In this article, we will provide a comprehensive review of the most relevant publications related to the use of plant extracts and natural compounds to prevent or ameliorate the effects of UVB irradiation on the skin.

## Plant extracts used against UVB-induced photodamage

**Fig. 2:**
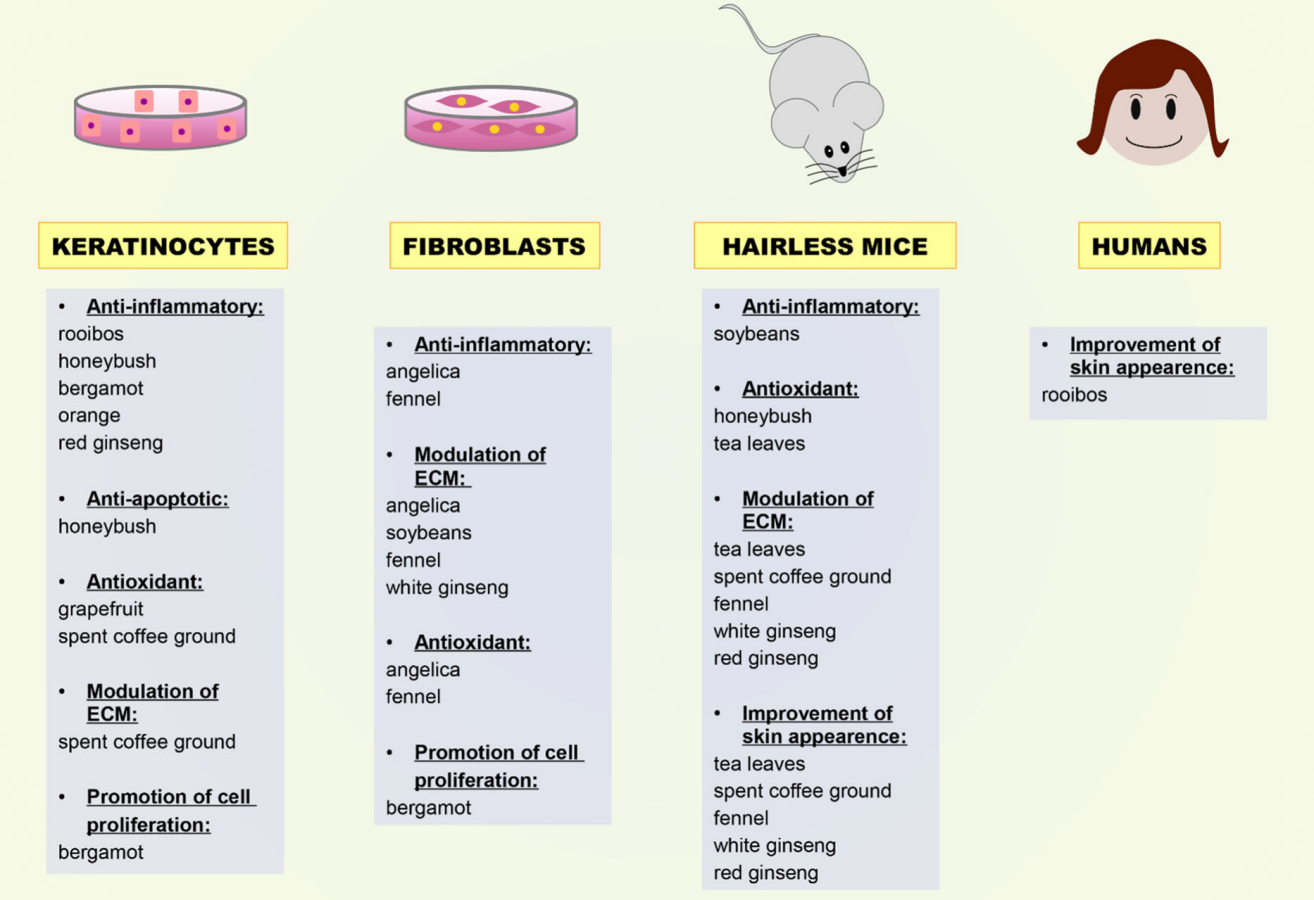
Summary of the main activities described for plant extracts against UVB-induced damage in different models

In this review, the most important and recent studies concerning plant species with anti-photoaging activities are described along with information on pharmacological effects of the relevant extracts against molecular processes driving skin aging in cellular and animal models. The scientific and common names, part of the plants used in the studies, identified bioactives as well as the pharmacological activities of the plants discussed in the text are summarized in Table 1. The results obtained in different experimental model systems of photoaging are summarized in Fig. 2.

**Table 1:** Plant extracts used against UVB-induced photoaging

Scientific and common names, part of the plants used in the studies, identified bioactives and main pharmacological activities of the plants

### Garden angelica (*Angelica archangelia)*

Common or garden angelica (*Angelica archangelia*) belongs to the Apiaceae family and is commonly cultivated due to its edible stems and roots. It has also been widely used in traditional medicine by virtue of its properties against anxiety and dementia and by its ability to induce hair growth (Sun et al. 2016a). The main compound of *Angelica archangelia* (AA) root crude extract is chlorogenic acid (Sun et al. 2016a). Treatment of normal human dermal fibroblasts (NHDF) with crude AA extract immediately after UVB irradiation was shown to decrease intracellular reactive oxygen species (ROS) production in comparison with non-treated irradiated cells (Sun et al. 2016a). AA root extract prevented UVB-induced damage of extracellular matrix (ECM) through blockage of collagen degradation and induction of collagen production, as treatment of NHDF with this extract restrained the phosphorylation of MAPK/AP-1 family members and activated TGF-β signaling. As a consequence, AA extract inhibited UVB-induced production and activity of matrix metalloproteases MMP-1 and MMP-3, restored procollagen type 1 synthesis and decreased the levels of the pro-inflammatory cytokine IL-6 in NHDF (Sun et al. 2016a).

#### Rooibos (*Aspalathus linearis*)

Rooibos or red bush is a broom-like member of the Fabaceae family, the leaves and twigs of which are rich in the polyphenol aspalathin (Magcwebeba et al. 2016b, a). Polyphenols are antioxidant molecules and are able to prevent excessive oxidative stress generated by accumulation of ROS (Saric and Sivamani 2016). Rooibos is originally from South Africa and has been traditionally used in treatment of skin diseases due to its antioxidant and anti-inflammatory properties (Magcwebeba et al. 2016b). Rooibos extract has also been used in antiaging cosmetics and treatment of patients with this formulation for 4 weeks was shown to decrease skin roughness and to improve the aspect of wrinkles (Gaspar et al. 2008). In an in vitro keratinocyte inflammatory UVB model, rooibos methanolic and aqueous extracts were shown to prevent accumulation of photodamage in HaCaT keratinocytes through indirect modulation of the inflammatory response (Magcwebeba et al. 2016b). Briefly, keratinocytes were exposed to UVB and further cultivated in the presence of the two different types of rooibos extract in different concentrations for 24 h. Results showed that both extracts were able to inhibit UVB-induced accumulation of pro-inflammatory intracellular IL-1α (icIL-1α) and to promote enhancement in the elimination of cells with increased levels of icIL-1α via apoptosis, protecting the cells against the cumulative damage caused by UVB (Magcwebeba et al. 2016b). In addition, a study was performed to test the anti-wrinkle properties of a cosmetic formulation containing a mixture of rooibos and tea extract (0.15% w/w) in middle-aged healthy women (Chuarienthong et al. 2010). Following application of the formulation twice a day during 28 consecutive days, parameters such as skin hydration and appearance of wrinkles were measured and the results were compared to the correspondent body areas tested before the treatment. The formulation containing rooibos and tea extracts was able to significantly decrease tissue wrinkles and slightly increase skin smoothness suggesting a potential role of rooibos extract to be used in cosmetic preparations (Chuarienthong et al. 2010).

#### Honeybush (*Cyclopia* spp.)

Honeybush is a group of leguminous plants that belong to the genus *Cyclopia* of the Fabaceae family. Honeybush plants grow in South Africa and their leaves, flowers and stems are commonly used for the preparation of teas and have been used in traditional medicine against skin disorders due to the high level of xanthones and flavanones (Gerber et al. 2015). The main bioactive compounds of honeybush are the xanthone mangiferin and the flavone hesperidin, which are polyphenols with high anti-inflammatory and antioxidant properties (Magcwebeba et al. 2016b). Topical administration of crude non-fermented and fermented Honeybush extract, but not of pure mangiferin and hesperidin, were able to inhibit UVB-induced edema in a study performed with hairless mice (Saric and Sivamani 2016). Furthermore, in the same study referred above for rooibos extract, aqueous honeybush extract was shown to have cytoprotective effects against UVB-induced oxidative stress (Magcwebeba et al. 2016b). In addition, treatment of HaCaT keratinocytes with honeybush methanol extracts after UVB treatment inhibited the accumulation of icIL-1α and reduced caspase-3 activity, consequently preventing apoptotic cell death (Magcwebeba et al. 2016b).

#### Tea plant (*Camellia sinensis*)

Tea is one of the most popular beverages in the world. White, green and black teas are products of the leaves, buds, and seeds from the tea plant *Camellia sinensis*, and differ in the way they are obtained and processed and, consequently, in the availability of bioactive compounds (Bosch et al. 2015). White tea is composed of young non-fermented leaves (Kim et al. 2015). Green tea is obtained by pan frying or steaming of leaves, a process that inactivates the endogenous polyphenol oxidase and increases the amount of caffeine (Lim et al. 2014; Lee et al. 2014a). Black tea is produced through fermentation/oxidation of the leaves and displays a high content of caffeine and tanins as well as of more complex polyphenols such as thearubigins and theaflavins which give it the brownish-red color (Kim et al. 2015).

The anti-photoaging effects of tea from *Camellia sinensis* have been proven in many studies (Suggs et al. 2014; Lim et al. 2014; Lee et al. 2014a; Kim et al. 2015). Lee and colleagues (2014) demonstrated that topical application of tea leaves extract to mice exposed to UVB ameliorated the appearance of wrinkles, decreased epidermal thickness, and reduced the expression and activity of MMP-3, leading to improvement of the ECM in comparison with UVB-irradiated non-treated animals (Lee et al. 2014a). In addition, this study showed that white tea and black tea, in general, were more efficient than green tea in preventing UVB-induced photoaging.

Oral administration of green tea seed extract (GTSE) to UVB-treated hairless mice decreased epidermal thickness allowing better skin hydration and recovered collagen density in a dose dependent manner (Lim et al. 2014). Results of treated animals revealed that GTSE, which contains catechins, gallic acid, caffeic acid and coumaric acid as its main bioactive compounds, is able to recover the activity of antioxidant enzymes, such as superoxide dismutase, catalase and glutathione peroxidase in a dose-dependent manner, in comparison to the skin of irradiated animals that were not treated with the extract. Finally, it was shown that GTSE decreased UVB-induced expression of MMP-1, MMP-3 and MMP-9 as well as increased the synthesis of collagen type 1 (Lim et al. 2014).

Altogether these data suggest the potential use of tea extracts in cosmetic formulations and as food supplements to be used against the damaging effects caused by UVB.

#### Bergamot (*Citrus bergamia*)

The protective effect of bergamot a polyphenol fraction (BPF) against UVB-induced damage of keratinocytes was studied by a group headed by Nisticò (Nisticò et al. 2015). It was shown that cultivation of HaCaT keratinocytes in the presence of BPF after UVB treatment leads to recovery of cell viability through modulation of the pro-inflammatory cytokine IL-1β via antioxidant mechanisms. Furthermore, treatment of cells with BPF was able to restore telomere length and telomerase activity in cells that received UVB irradiation suggesting that BPF has the ability to modulate signal transduction pathways related to immunoregulation and cell proliferation (Nisticò et al. 2015).

#### Grapefruit (*Citrus x paradisi*)

Grapefruit, a hybrid plant that belongs to the Rutaceae family originally from Central and South America is commonly used in general alimentation as well as in cosmetic formulations due to its vivid color and citric scent. Grapefruit is rich in phenolic compounds, among which gallic acid (GA) is the most abundant (Nobile et al. 2016). In a study conducted by Pérez-Sánchez and colleagues cultivation of human keratinocytes, prior to UVB irradiation, in the presence of grapefruit extract alone (12.5–100 µg/mL) or in combination with rosemary extract (12.5–100 µg/mL), which is also rich in GA, was investigated. Results demonstrated that grapefruit extract alone or in combination with rosemary extract is able to improve cell viability and to decrease UVB-induced intracellular ROS levels in a dose-dependent manner in comparison to irradiated non-treated cells (Pérez-Sánchez et al. 2014). These results suggest the potential use of grapefruit extract as an antioxidant in cosmetic formulations.

#### Orange (*Citrus sinensis*)

Citrus fruit peels, especially orange peel, have been used in traditional medicine due to their beneficial effects against digestive and respiratory problems (Yoshizaki et al. 2014). Extracts of orange peel contain up to 90% polymethoxyflavonoids, among which tangeretin, nobiletin and 3,3′;4′,5,6,7,8-heptamethoxyflavon are the most abundant. In a study in which the protective effects of orange peel (OP) against photodamage were investigated, Yoshizaki and colleagues (2014) observed that treatment of HaCaT keratinocytes with extract of OP prior to UVB irradiation was able to modulate UVB-induced inflammatory response by suppression of cyclooxygenase (COX)-2 expression and prostaglandin (PG) E2 production via PPAR-γ activation (Yoshizaki et al. 2014).

#### Spent coffee ground/coffee (*Coffea* spp.)

Together with tea, instant coffee has been one of the most widely consumed beverages in the world for decades (Choi et al. 2016). For the preparation of instant coffee, roasted and grounded beans are submitted to thermal water extraction, a process that results in the production of a residue called spent coffee ground (SCG). This residue is mainly composed of insoluble solids and components that are not completely extracted during thermal infusion, such as caffeine and chlorogenic acid (Choi et al. 2015). SCG has antioxidant properties among other functions (Choi et al. 2016) The potential role of ethanolic SCG (ESCG) extract against skin damage caused by UVB was investigated in HaCaT keratinocytes and in the hairless mouse model (Choi et al. 2015, Choi et al. 2016). Results revealed that ESCG extract contains a higher level of caffeine than chlorogenic acid and treatment of UVB-irradiated HaCaT keratinocytes with 1, 10, 25 and 50 µg/mL ESCG for 1 h before irradiation decreased intracellular ROS production in a dose-dependent manner (Choi et al. 2015). ESCG extract, when orally administered to hairless mice in the course of UVB treatment (12 weeks), decreased the ratio of wrinkle area in comparison to the skin of irradiated animals that were not fed with the extract (Choi et al. 2015).

In another study, the same group showed that topical application of an oil fraction (OSCG) and an ethanolic extract (ESCG) of SCG added to a basic cosmetic formulation led to a decrease in the wrinkle area (Choi et al. 2015), in a similar manner as observed for oral administration in the hairless mouse model before (Choi et al. 2015). Wrinkle improvement of SCG treated animals was due to the ability of this extract to reduce UVB-induced epidermal thickness, increase water holding capacity and decrease erythema area (Choi et al. 2016). Topical application of OSCG and ESCG in the dorsal area of UVB-irradiated mice also inhibited MMP-2 expression and enhanced collagen-1 production, leading to recovery of ECM structure. In addition, treatment of UVB-irradiated HaCaT keratinocytes with OSCG decreased UVB-induced intracellular ROS production, and decreased MMP-2 and MMP-9 expression in comparison with irradiated control cells (Choi et al. 2016).

#### Soybeans (*Glycine max*)

Soybeans are an important plant source of protein. Effects of soybean against photodamage were reported through increase of collagen and hyaluronan synthesis, reduction of MMP expression, and stimulation of fibroblast proliferation (Lee et al. 2014b). The anti-inflammatory effect of a fermented soybean extract against the damage caused by UVB irradiation were investigated in co-cultures of NHDF and keratinocytes exposed to a sub-erythemal dose of UVB (Lee et al. 2014b). Treatment of co-cultures with 10 µM genistein, the main compound found in soybeans, slightly decreased the levels of the pro-inflammatory cytokine IL-6 and MAPK signaling, through inhibition of phosphorylation of p38, ERK, and JNK in comparison to UVB irradiated control cells (Lee et al. 2014b). Dietary supplementation with fermented soybean extract reduced UVB-induced epidermal thickening of hairless mice submitted to mild UVB treatment. In the same model, fermented soybean diet was able to modulate the inflammatory response elicited by UVB irradiation through decreasing the number of infiltrating macrophages as well as by reducing the expression of iNOS and COX-2 enzymes, which work as regulators of pro-inflammatory mediators released in response to photodamage (Lee et al. 2014b).

#### Fennel (*Foeniculum vulgare* Mill.)

Fennel is a highly aromatic and flavorful herb. Besides its culinary use, it is commonly used in traditional medicine in the form of teas due to its antioxidant and anti-inflammatory properties (Sun et al. 2016b). Fennel seed extracts are rich in cholorogenic acid, ferrulic acid and rutin. In a study performed by Sun and colleagues (2016) the anti-photoaging properties of *Foeniculum vulgare* (FV) were evaluated in NHDF as well as in the hairless mouse model (Sun et al. 2016b). For this purpose, extract of dried FV seeds was added to cell culture medium of NHDF immediately after UVB-irradiation. Cells were kept in the presence of the extract in different concentrations (1, 10 and 100 µg/mL) for 72 h and then analyzed for photoaging-related parameters. Treatment of cells with a FV extract was able to attenuate UVB-induced intracellular ROS production and to increase the level of the antioxidant glutathione (GSH). FV extract employment promoted enhancement of Nrf2 nuclear translocation in UVB-irradiated fibroblasts in comparison to vehicle-treated cells. In addition, cultivation of NHDF in the presence of a FV seed extract efficiently decreased the expression and activity of UVB-induced metalloproteases, decreased the levels of the pro-inflammatory cytokine IL-6 and promoted increased transcription of procollagen type 1 through TGF-β signaling activation (Sun et al. 2016b).

In a similar way, oral administration of FV to UVB-irradiated hairless mice efficiently improved the appearance of wrinkles and decreased the epidermal thickness in comparison with irradiated mice fed with vehicle. This effect was due to the ability of FV to inhibit UVB-induced ECM degradation through suppression of MMP-1 activity and to recover ECM density through increment of procollagen type 1 and elastin production via TGF-β activation (Sun et al. 2016b).

#### Ginseng (*Panax ginseng*)

Korean ginseng is a well-known medicinal herb that has been widely used in traditional Eastern medicine to treat various diseases due to its broad range of biological activities, including anti-inflammatory, antioxidant, anti-tumoral and anti-stress effects (Hwang et al. 2014a). Ginsenosides and saponins are the main components responsible for the biological activities of ginseng (Hwang et al. 2012, 2014a). Ginseng can be classified as white ginseng (WG) or red ginseng (RG), according to processing conditions, and each of them is presumed to have different bioactive compounds (Lim et al. 2015). WG is made by peeling the fresh ginseng roots and sun-drying them without the following steaming process. RG is made by steaming and drying the fresh ginseng, a process that is believed to enhance its bioactivity (Hwang et al. 2013; Lim et al. 2015; Saewan and Jimtaisong 2015). Both extracts are rich in ginsenosides and saponins.

Treatment of UVB-irradiated NHDF with an extract of enzyme-modified white ginseng (EMWG) was shown to suppress MMP-1 production in comparison to irradiated control cells. In addition, cultivation of UVB-treated fibroblasts in the presence of its main component, ginsenoside F2 alone, was able to increase procollagen type 1 production and to decrease MMP-1 secretion (Hwang et al. 2014a). In hairless mice, topical application of EMWG and with pure ginsenoside F2, after UVB treatment prevented degradation of ECM components through inhibition of MMP-1 and increased the density of collagen fibers through activation of procollagen I production and TGF-β activation (Hwang et al. 2014a).

The antiaging properties of an extract of enzyme-modified red ginseng (EMRG) were tested in murine keratinocytes (Chung et al. 2015) and in the hairless mouse model (Hwang et al. 2013). Sub-erythemal doses of UVB treatment with the saponin ginsenoside-Rh3 derived from EMRG decreased the expression of the granulocyte macrophage colony stimulating factor (GM-CSF), inhibiting UVB-induced activation of the inflammatory response in keratinocytes (Chung et al. 2015). In the hairless mouse model, oral administration of EMRG led to reduction of UVB-induced wrinkle area, epidermal thickening and skin dryness. EMRG treatment modulated ECM by promotion of procollagen type 1 synthesis, enhancement of TGF-β1 secretion and attenuation of MMP-1 levels in comparison to UVB-irradiated animals fed with vehicle (Hwang et al. 2013). In addition, treatment of irradiated mice with EMRG protected the skin homeostasis by increasing the expression of profilaggrin and filaggrin (Hwang et al. 2013).

## Natural compounds used against photoaging

**Fig. 3:**
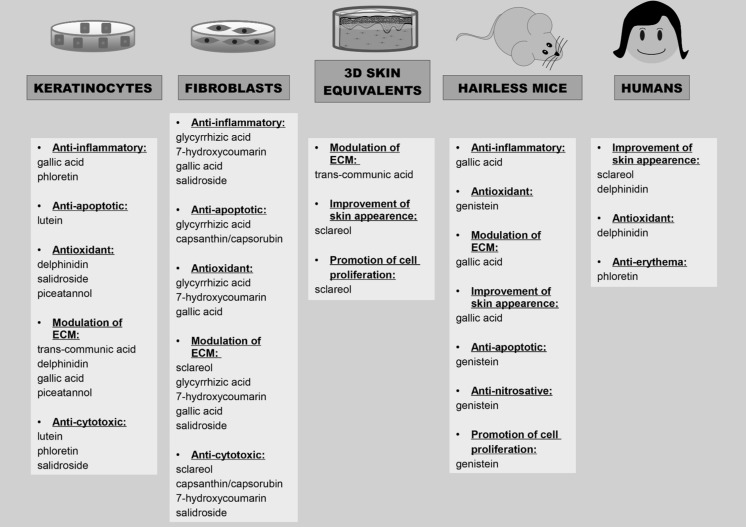
Summary of the main activities described for natural compounds against UVB-induced damage in different models

As outlined above, several plant extracts with pharmacological activity against UVB-induced photoaging have been identified. Subsequent studies aimed to identify the active principle (s) in a given extract, which can be either used instead of the plant extract or, more frequently, as a means to standardize the production of active extracts with similar activity. The natural compounds fall primarily in two classes, namely isoprenoids (Table 2) and phenolic compounds (Table 3), which are described below. The results obtained with natural compounds in different model systems are summarized in Fig. 3.

**Table 2:** Pharmacological activities and described effective dosage (IC 50) of natural isoprenoids effective against UVB-induced photoaging

**Table 3:** Pharmacological activities and described effective dosage (IC 50) of natural phenolic compounds used against UVB-induced photoaging

### Studies with isoprenoids

#### Sclareol

Sclareol is an isoprenoid isolated from common sage (*Salvia officinalis)* which is widely used as natural fragrance. This natural compound exhibits diverse biological properties such as antioxidative, anti-fungal, anticancer, antimicrobial, anti-inflammatory, and anticholinesterase activities (Park et al. 2016). Park and colleagues (2016) used human dermal fibroblasts and reconstructed human skin to investigate the efficacy of sclareol to prevent the damage caused by UVB irradiation and conducted a clinical study to identify its ability to ameliorate the signs of photoaging of human skin (Park et al. 2016). In this study they observed that sclareol is able to recover the proliferative capacity of Hs68 cells treated with UVB while it inhibits the UVB-induced expression of MMPs via regulation of AP-1 constituents (Park et al. 2016). Reconstructed human skin models exposed to 3 UVB irradiations and subsequently treated with two different concentrations of sclareol (1 and 10 µM) exhibited decreased epidermal thickness and recovery of cellular proliferative capacity in comparison to UVB-irradiated 3D models that were not treated with the compound. In addition, in a clinical trial, the study investigated the effect of a cosmetic formulation containing 0.02% sclareol on facial wrinkle appearance was investigated. After 12 weeks of treatment, total wrinkle area, percentage of wrinkle area, and total wrinkle length were significantly reduced in the test group compared to the control group suggesting that sclareol may be an effective cosmetic ingredient to help reducing the effects of UVB-induced photoaging (Park et al. 2016).

#### Trans-communic acid (TCA)

Japanese red pine (*Pinus densiflora*) is a popular ornamental pine that has long been cultivated in Asian countries (Huh et al. 2015). The green needle-like leaves of this tree have historically been used as culinary color and flavor ingredients as well as in traditional medicine against inflammatory diseases. Shedded pine leaves keep the green color for about two years and after this period they turn brown, a process that leads to increased abundance and availability of trans-communic acid (TCA) (Huh et al. 2015). TCA, a diterpene with a labdene skeleton, is active against microorganisms and has antitumoral and antioxidant properties (Huh et al. 2015). Huh and colleagues (2015) investigated the protective effect of brown pine leaf extract (BPLE) and TCA against the effects of UVB irradiation in HaCaT keratinocytes and in human reconstructed skin models (Huh et al. 2015). Treatment of HaCaT keratinocytes with BPLE and TCA prior to UVB irradiation promoted inhibition of UVB-induced MMP-1 expression and AP-1 transactivation in a dose dependent manner (Huh et al. 2015). TCA was shown to be the active constituent against MMP-1 expression in BPLE and both TCA and BPLE attenuated UVB-induced Akt phosphorylation through direct inhibition of PI3 K activity. Furthermore these compounds were able to reduce collagen degradation and mitigate MMP-1 expression in a human skin equivalent model (Huh et al. 2015). Altogether these results suggest TCA as a potential ingredient for cosmeceutical preparations.

#### Glycyrrhizic acid (Gl.A)

Glycyrrhizic acid, a triterpene saponin and component of licorice roots has been reported to exhibit anti-inflammatory, antioxidant, and cardio-, neuro- and hepatoprotective properties (Afnan et al. 2012; Farrukh et al. 2015). Licorice extracts have also been historically used in the treatment of skin disorders as well as in make-up and skincare products (Afnan et al. 2012). The inhibitory effect of Gl.A against UVB-induced photoaging of NHDF and the molecular mechanisms involved in this process were described by Afnan and colleagues(2012). For this study Hs68 NHDF were cultivated in media containing Gl.A (5–50 µM) for 24 h before and after a single dose of irradiation (5−20 mJ/cm^2^). It was observed that Gl.A enhances cell viability and prevents UVB-induced nuclear and cytoplasmic morphological alterations in a dose-dependent manner. Cultivation of NHDF in media containing Gl.A. inhibited UVB-induced ROS accumulation and NF-κB activation. These events led to inhibition of MMP-1 and hyaluronidase activation, preventing general ECM degradation and induction of apoptosis via caspase-3 (Afnan et al. 2012). Furthermore, in a more recent publication, Gl.A treatment was shown to inhibit UVB induced endoplasmic reticulum stress in Hs68 cells and was able to restore intracellular Ca^2+^ imbalance caused by irradiation (Farrukh et al. 2015). Gl.A also acted over MAPK signaling pathway, since pretreatment of Hs68 cells with 10 or 25 µM of this compound significantly reduced the UVB induced phosphorylation of p38, JNK and pMEK. Altogether these data suggest that Gl.A is a potential candidate to be used in cosmetic products due to its anti-oxidant and anti-inflammatory activities (Farrukh et al. 2015).

#### Capsanthin and capsorubin

Capsanthin and capsorubin are carotenoids found exclusively in red pepper fruits (Fernández-García et al. 2016). Carotenoids are tetraterpenes which present high antioxidant capacity, in particular quenching of singlet molecular oxygen and scavenging of peroxyl radicals (Bosch et al. 2015).

The protective properties of capsanthin and capsorubin against UVB-induced photodamage of CCD-1064Sk human dermal fibroblasts were evaluated (Fernández-García et al. 2016). For this purpose, cells were preincubated with 1 µM of capsanthin or capsorubin prior to UVB treatment and analyzed for DNA damage and induction of apoptosis. Results demonstrated that the two compounds are able to protect NHDF from UVB-induced cytotoxicity. In comparison to vehicle-treated cells, treatment with capsanthin or capsorubin decreased the number of DNA strand breaks, protecting the cells from excessive DNA damage. Moreover, cultivation of NHDF in media containing capsanthin prior to UVB irradiation prevented caspase-3 cleavage and consequently apoptotic cell death. Collectively, these results reinforce the idea that these compounds could be used as dietary supplement to improve natural photoprotection (Fernández-García et al. 2016).

#### Lutein

Lutein is a carotenoid present in many vegetables and fruits as well as in yellow silk cocoons. Carotenoids are of great importance for human health as they are present in high concentrations in human retina, where they work as antioxidants that protect human eyes from the damage caused by sunlight (Pongcharoen et al. 2013). The protective effect of different doses of silk lutein against UVB-induced cell death and cell cycle arrest was investigated in a keratinocyte cell line (CCD 1102 KERTr) and in primary human keratinocytes (Pongcharoen et al. 2013). Cultivation of keratinocytes for 24 h in media containing silk lutein (5 and 15 µM) before irradiation protected them from UVB-mediated cell damage as cells submitted to this treatment remained viable after receiving 4 mJ or 16 mJ of UVB irradiation. Silk lutein pretreatment in these same concentrations also decreased the number of apoptotic cells in comparison with UVB-irradiated cells cultivated in normal media and in media containing commercially available plant-derived lutein, suggesting that this compound may have a significant benefit in terms of skin protection against UVB-induced photoaging. Nonetheless no significant effect was observed in cell proliferation of irradiated cells cultivated in the presence or absence of lutein in comparison to control non-irradiated cells, suggesting that in these concentrations (5 and 15 µM) lutein is not able to stimulate keratinocyte proliferation, or that rate of proliferation is not enough to overcome apoptotic cell death in these conditions (Pongcharoen et al. 2013).

### Studies with phenolic compounds

#### Gallic acid (GA)

Gallic acid, a phenolic compound naturally found in green tea leaves, as well as in other natural sources, protects cells and tissues against oxidative damage and is able to modulate the inflammatory response (Hwang et al. 2014b). Using NHDF and mouse models, Hwang and colleagues (2014b) investigated the regenerative properties of GA administration against photodamage caused by UVB irradiation. Treatment of NHDF with 1 and 10 µM GA immediately after UVB treatment (144 mJ/cm^2^) was able to decrease cytoplasmic ROS production by 10% and 45%, and to lower levels of MMP-1 secretion and IL-6 production by 56% and 48%, respectively, in comparison to irradiated cells not treated with GA. These effects were mainly due to GA’s ability to decrease UVB-induced phosphorylation of AP-1 transcription factor proteins c-Jun and c-Fos. Topical application of GA in mice submitted to repeated sub-erythemal doses of UVB improved the appearance of wrinkles and general skin hydration in comparison with control UVB-treated mice. Furthermore, histological analysis demonstrated that GA was able to inhibit UVB-induced epidermis thickening and collagen degradation in the upper dermis. Prevention of collagen breakdown in this model was dependent on GA’s ability to block MMP-1 and IL-6 expression and activity while increasing TGF-β, elastin and pro-collagen I expression (Hwang et al. 2014b).

These results were reinforced by another study, in which Sun and colleagues (2015) evaluated the anti-photoaging potential of *Galla chinensis* (GAC) extract in vitro and in vivo (Sun et al. 2015). GAC is a traditional natural compound available in galls formed upon the installation of aphid parasites in the leaves of the Anachardiacea family of plants, and GA is one of its main compounds. GAC has been used in Chinese traditional medicine for many centuries and has been reported to possess antibacterial, antiviral, and antifungal activities. Administration of GAC (1 and 10 µg/mL) subsequently to UVB treatment was able to decrease ROS production of NHDF by around 28 and 46%, respectively. Treatment of UVB-irradiated NHDF and HaCaT keratinocytes with GAC was able to decrease MMP-1 and IL-6 production and activity in a similar kinetics as observed before by Hwang and colleagues (2014b). In a comparable way, topically applied and orally administrated GAC extracts were able to reduce UVB-induced epidermal thickness and to restore extracellular matrix density via TGF-β activation of elastin and procollagen type 1 synthesis in the hairless mouse model (Sun et al. 2015). Altogether these data suggest that GA is a valuable ingredient to be used as food supplement as well as in cosmetic preparations against photoaging caused by UVB.

#### 7-Hydroxycoumarin (7-OHC)

Coumarin is a naturally occurring phytoconstituent with anticoagulant, antimutagenic and antitumorigenic properties ubiquitously distributed throughout the plant kingdom (Karthikeyan et al. 2016). 7-OHC was reported to present a high SPF value (Sun Protection Factor, which represents the fraction of total sunburn that is reduced by a compound) and treatment of human dermal fibroblast with 30 µM 7-OHC prior to single acute UVB treatment significantly prevented UVB-induced cytotoxicity in this cell type (Karthikeyan et al. 2016). 7-OHC photoprotective effects were shown to be mediated by its ability of restoring the antioxidant activity and by modulating the expression of inflammatory molecules such as NF-κB as well as by decreasing the mRNA expression of matrix MMPs such as MMP-1 and MMP-9 after UVB-exposure (Karthikeyan et al. 2016).

#### Delphinidin

Delphinidin is a primary plant pigment that gives blue hues to some flowers and fruits, and presents antioxidant properties. This aglycon form of anthocyanin was already reported to have potent inhibitory effect against oxidative-stress and UVB-induced skin damage (Allemann and Baumann 2009; Saewan and Jimtaisong 2015). In a recent study conducted by Sobiepanek and colleagues (2016), the influence of delphinidin in modulating the mechanical properties of keratinocytes in response to UVB treatment was investigated (Sobiepanek et al. 2016). It was demonstrated that pre and post treatment of HaCaT keratinocytes with non-cytotoxic concentrations (5 or 10 µM) of delphinidin were able to abolish the deleterious effect of UVB irradiation in the cell elastic modulus as well as to restore the metabolic activity of these cells. Restoration of cell stiffness was more prominent in cells that received delphinidin treatment after UVB irradiation suggesting that the regenerative effects of this compound might be due to antioxidant and inhibitory effects on MMPs activation (Sobiepanek et al. 2016).

#### Genistein

Genistein is an isoflavone that occurs in soybeans which has been reported to have anti-photoaging and anti-photocarcinogenesis activity (Wang et al. 2010; Lee et al. 2014b; Terra et al. 2015). The effect of genistein treatment against photodamage was investigated by Terra and colleagues (2015) in the hairless mouse model. In this study it was shown that intraperitoneal administered genistein (10 mg/kg) is able to protect against UVB-induced lipid peroxidation but does not prevent inhibition of catalase activity in these conditions. Furthermore, 10 mg/kg genistein exhibited anti-nitrosative activity and inhibited UVB-induced ONOO^−^ formation in hairless mice. This compound was able to confer skin protection and enhance cell proliferation through decreased expression of p53 and also reduced levels of apoptosis in this model (Terra et al. 2015).

#### Phloretin

Phloretin is an almost water-insoluble dihydrochalcone found in apple and apple juice. To increase the solubility of this compound in order to investigate its anti-photoaging properties, Shin and colleagues (2014) generated a sulfonated form of phloretin. Treatment of HaCaT keratinocytes with phloretin dissulfonate (PS) for 12 h after UVB irradiation recovered cell viability in a dose dependent manner. Administration of PS (50–200 µg/mL) resulted in reduced DNA damage, attenuated pyrimidine dimer (CPD) formation and increased the number of p-p53 and γH2A.X positive cells compared to untreated cells exposed to UVB. Investigation of the effect of PS on the modulation of nucleotide excision repair (NER) genes upon UVB irradiation revealed that PS might enhance the repair of UVB-induced DNA damage in HaCaT keratinocytes through increased expression of the XPA and XPC genes. In addition, PS was able to inhibit UVB-induced release of the inflammatory mediators IL-6 and prostaglandin-E2. In human skin exposed to minimal erythemal dose (MED) of UVB, pre-treatment with phloretin was able to reduce erythematous area, suggesting a potential use of this compound in skin photoprotection (Shin et al. 2014).

#### Salidroside

Salidroside is a phenylethanoid found in *Rhodiola rosea L.*, a plant that grows in high-altitude regions and has long been used as traditional medicine in China and Eastern Europe due to its beneficial effects against multiple pathological processes such as cerebral and endothelial dysfunction, hepatic injury and osteoporosis (Mao et al. 2015). The anti-photoaging properties of this compound were investigated by Mao and colleagues (2015) using UVB-irradiated NHDF. According to this study, pre-treatment of NHDF with 1-10 µM salidroside was able to recover cell viability and decrease the number of Senescence-Associated-β-Galactosidase (SA-β-Gal) positive cells in a dose-dependent manner after UVB irradiation. Salidroside treatment prevented UVB-induced cell cycle arrest and decreased the expression of the senescence-associated factors p21, p53 and p16. In addition, salidroside was able to protect the cells from UVB-induced synthesis of MMP-1 and of the pro-inflammatory cytokines IL-6 and TNF-α (Mao et al. 2015). More recently, Xiao-Ying and colleagues (2016) showed that salidroside treatment is also able to protect HaCaT keratinocytes against UVB-mediated decrease in cell viability in a dose-dependent manner. In this study, they also showed that salidroside displays anti-oxidant capacity since pre-treatment of HaCaT keratinocytes with 20 or 40 µg/mL salidroside was able to decrease UVB-induced cytoplasmic ROS levels as well as recover transcription activity of *Nrf2* and increase gene and protein expression of its downstream antioxidants NQO1 and HO-1. In addition, using guinea pigs, this study demonstrated that oral administration of salidroside (0.1% w/w) prior to UVB irradiation prevents thickening of the epidermis and inhibits UVB-induced apoptosis and the appearance of sunburn cells (SBC) in the epidermis (Xiao-Ying et al. 2016). Overall these results suggest the potential role of salidroside as a protective compound against UVB-induced senescence.

#### Piceatannol

Piceatannol is known as strong antioxidant present in the seeds of passion fruit (*Passiflora edulis*). Administration of piceatannol to normal human keratinocytes prior to UVB treatment was shown to decrease cytoplasmic ROS production in a dose dependent manner (Maruki-Uchida et al. 2013). In NHDF, piceatannol treatment decreased UVB-induced MMP-1 activity suggesting its potential use in photoprotective cosmetic preparations (Maruki-Uchida et al. 2013).

## Conclusion

Plant extracts and natural compounds are traditionally used in the treatment of skin diseases as well as in rejuvenating and photoprotective cosmetic formulations. The growing demand in the consumer market for natural cosmetics justifies the evaluation of the bioactive properties and the efficacy of these natural products in combating the damaging effects caused by sunlight. In this work we provide a comprehensive review of the most relevant and recent publications that evaluate the performances and effects of plant extracts and constituents against UVB-induced damage in several experimental models of photoaging. As shown above, much has been researched on the various properties exhibited by different plant extracts and isolated natural compounds. Most of them appear to exhibit antioxidant activities, probably due to the adaptive ability of plants to survive in a highly oxidizing environment, but also anti-inflammatory, anti-apoptotic, anti-cytotoxic, pro-synthetic and pro-proliferative activities which, together, have the potential to help diminish or protect the skin from the deleterious effects of UVB radiation. Much research is still required on the subject both to enable the extrapolation of the results obtained in models for the adequate treatment of human skin as well as to understand the molecular mechanisms involved in these processes and to design formulations that contain not only effective concentrations of extracts or their bioactive constituents but also allow them to remain in their active form following oral or topical application.

## Abbreviations

Akt: Protein kinase B
AP-1: Activator protein 1
COX-2: Cyclooxygenase-2
ECM: Extracellular matrix
ERK: Extracellular signal-regulated kinase
GSH: Glutathione
icIL-1α: Intracellular interleukin-1 Alpha
IL-1β: Interleukin-1 Beta
IL-6: Interleukin-6
iNOS: Inducible-nitric oxide synthase
JNK: C-Jun N-terminal kinase
MAPK: Mitogen-activated protein kinase
MED: Minimal erythemal dose
MMPs: Matrix metalloproteases
NER: Nucleotide excision repair
NF-κB: Nuclear Factor kappa-light-chain-enhancer of activated B cells
NHDF: Normal human dermal fibroblasts
Nrf2: Nuclear factor (erythroid-derived 2)-Like 2
ONOO: Peroxynitrite
PG: Prostaglandin
PI3K: Phosphatidylinositol-4,5-bisphosphate 3-kinase
pMEK: Phosphorylated mitogen-activated protein kinase kinase
PPAR-γ: Peroxisome proliferator-activated receptor gamma
ROS: Reactive oxygen species
SASP: Senescence-associated secretory phenotype
SA-β-Gal: Senescence-associated beta galactosidase
SBC: Sunburn cells
TGF-β: Transforming growth factor beta
TNF-a: Tumor necrosis factor alpha
UV: Ultraviolet
UVB: Ultraviolet B
